# Anti-CD38/Anti-CD20 Rescue Therapy for Posttransplant Recurrent FSGS

**DOI:** 10.1016/j.ekir.2026.106668

**Published:** 2026-06-16

**Authors:** Juliette Leon, Elise Ducrocq, Marie-Camille Lafargue, Dominique Bertrand, Lucie Maigret, Mohamad Zaidan, Arthur Michon Colin, Eric Thervet, Antoine Durrbach, Nassim Kamar, Betoul Schvartz, Nicolas Bouvier, Manal Mazloum, Hamza Naciri Bennani, Manon Guezennec, Dalila Es-Saleh, Antoine Troger, Carole Burger, Marie-Bénédicte Le Stang, Camille Roger, Frank Hullekes, Olivier Aubert, Julien Zuber, Leonardo V. Riella, Vincent Audard, Dany Anglicheau

**Affiliations:** 1 Department of Kidney Diseases and Metabolism, Transplantation and Clinical Immunology, Necker Hospital, Assistance Publique–Hôpitaux de Paris, and Université Paris Cité, Paris, France; 2 Department of Nephrology, Transplantation and Hemodialysis, Rouen University Hospital, Rouen, France; 3 Department of Nephrology, Hypertension, Dialysis and Kidney Transplantation, Tours University Hospital, Tours, France; 4 Department of Nephrology, Dialysis, Transplantation, Bicêtre Hospital, Assistance Publique–Hôpitaux de Paris, Paris, France; 5 Department of Nephrology, Georges Pompidou European Hospital, Assistance Publique–Hôpitaux de Paris, and Université Paris Cité, Paris, France; 6 Department of Nephrology and Transplantation, AP-HP Henri-Mondor Hospital, Institut National de la Santé et de la Recherche Médicale (INSERM) U1186, Gustave Roussy Institute, Université Paris-Saclay, Créteil, France; 7 Department of Nephrology and Organ Transplantation, Axe TImE, Toulouse Rangueil University Hospital, Institut National de la Santé et de la Recherche Médicale (INSERM) UMR 1291, Toulouse Institute for Infectious and Inflammatory Diseases (Infinity), University Paul Sabatier, Toulouse, France; 8 Department of Nephrology, Dialysis and Transplantation, Reims University Hospital, Reims, France; 9 Department of Nephrology, Dialysis and Transplantation, Caen Normandie University Hospital, Caen, France; 10 Department of Nephrology, Dialysis and Transplantation, Lapeyronie University Hospital, Montpellier, France; 11 Department of Nephrology, Hemodialysis, Apheresis and Kidney Transplantation, Félix-Guyon University Hospital, Saint-Denis, La Réunion, France; 12 Department of Transplantation, Nephrology and Clinical Immunology, Hospices Civils de Lyon, Edouard Herriot University Hospital, Lyon, France; 13 Department of Surgery, Center for Transplantation Science, Massachusetts General Hospital, Harvard Medical School, Boston, Massachusetts, USA; 14 Nephrology and Renal Transplantation Department, Assistance Publique-Hôpitaux de Paris, French Rare Disease Center “Idiopathic Nephrotic Syndrome”, Henri Mondor University Hospital, Créteil, France; 15 Department of Nephrology and Transplantation, AP-HP Henri-Mondor Hospital, Université Paris Est Créteil, Institut National de la Santé et de la Recherche Médicale (INSERM), U955, Institut Mondor de Recherche Biomédicale, Créteil, France

**Keywords:** anti-CD38 antibody, antinephrin antibody, focal segmental glomerulosclerosis, kidney transplantation, recurrence glomerular disease

## Abstract

**Introduction:**

Recurrent focal segmental glomerulosclerosis (FSGS, rFSGS) is a severe complication after kidney transplantation, often resistant to standard therapies and requiring prolonged apheresis. Combined depletion of plasma cells (anti-CD38) and B cells (anti-CD20) has emerged as a potential strategy; however, multicenter real-world data integrating clinical, biological, and histological characterization remain limited.

**Methods:**

We retrospectively studied 17 kidney transplant recipients with rFSGS treated with daratumumab (anti-CD38) administered after and/or in combination with anti-CD20 therapy across 12 French centers. Patients were clinically and histologically characterized at treatment initiation. Clinical response, safety, and longitudinal antinephrin antibody trajectories were assessed.

**Results:**

Median time from recurrence to daratumumab initiation was 5 months. All patients had previously received B-cell-depleting therapy. After the first daratumumab course (1–8 injections), 7 patients (41%) achieved complete remission (CR) and 4 (24%) achieved partial remission (PR), allowing discontinuation of apheresis in all responders. Median response time was 24 days. During a median follow-up of 8 months, 3 responders relapsed but regained remission after retreatment. Median proteinuria decreased from 3.9 g/g to 1.4 g/g at 1 month (*P* = 0.029). Among 11 patients tested, 3 had positive antinephrin antibodies. Two patients showed marked posttreatment declines paralleling clinical response, whereas 1 did not. Treatment was well-tolerated.

**Conclusion:**

In this multicenter real-world cohort, combined plasma cell and B-cell depletion was associated with meaningful remission rates in refractory rFSGS and was accompanied by dynamic changes in antinephrin antibodies in selected cases. Prospective trials are warranted to define optimal patient selection and dosing, and to clarify its place in therapy.

## Graphical abstract

**Figure:**
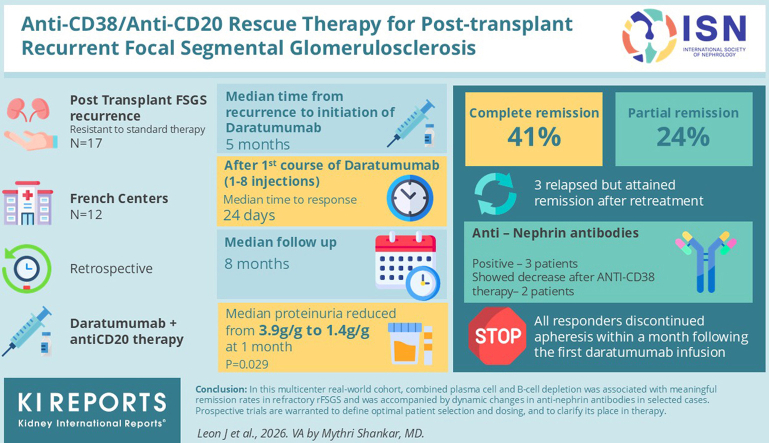

FSGS is one of the most frequent glomerulopathies^1^ and can progress to end-stage kidney disease.^2^ In patients with idiopathic FSGS where no secondary cause is identified, the risk of recurrence after kidney transplantation is high^3^ occurring in approximately one-third of cases. Recurrence markedly increases the risk of allograft loss and mortality.^3^, ^4^, ^5^

Current therapeutic strategies for rFSGS include combinations of corticosteroids, plasma exchange (PEX), immunoadsorption (IA), calcineurin inhibitor optimization, and anti-CD20 monoclonal antibodies.^6^ Unfortunately, despite aggressive management, a substantial proportion of patients fail to achieve sustained remission and long-term PEX or IA dependency markedly impairs quality of life.^3^^,^^7^

Although the pathophysiology of FSGS remains unclear, it is widely accepted that primary FSGS is driven by a circulating permeability factor that disrupts podocyte structure and function.^2^ Several observations support this hypothesis: (i) perfusion of isolated rat glomeruli with patient plasma induces proteinuria,^8^ (ii) transplacental transmission from mother to child during pregnancy has been documented,^9^ and (iii) a renal allograft dysfunctional due to fulminant rFSGS in one recipient could function normally in another recipient after retransplantation.^10^ The identity of this circulating factor remains unknown; however, the partial efficacy of B-cell–targeted therapies in some patients as well as the efficacy of IA suggest an antibody-mediated mechanism.^11^^,^^12^ Recent studies have identified autoantibodies against nephrin, a key podocyte protein, in patients with both primary FSGS nd rFSGS^13^^,^^14^ which correlate with disease activity.

Following this hypothesis, current therapies targeting B cells may not fully address ongoing antibody production by long-lived plasma cells. Daratumumab, a monoclonal antibody against CD38 expressed on plasma cells, has recently been reported to induce remission, often in combination with anti-CD20 antibodies, in cases of steroid-resistant FSGS in native kidneys^15^^,^^16^ and in rFSGS.^17^, ^18^, ^19^, ^20^ However, data remain limited, particularly regarding biological and histological correlates of response, therapeutic sequencing, and real-world implementation across centers.

The aim of the present study was to assess the clinical outcomes and safety of combined plasma cell– and B-cell–depleting therapy in a multicenter real-world cohort of patients with rFSGS, and to investigate longitudinal antinephrin antibody dynamics as exploratory biomarkers of response.

## Methods

### Source Population and Study Design

We identified, through a nationwide survey, all adult kidney transplant recipients diagnosed with rFSGS after transplantation and subsequently treated with daratumumab (anti-CD38). Seventeen patients were identified across 12 French transplant centers. All identified patients had also received anti-CD20 therapy, either before or concomitantly with daratumumab, allowing analysis of a combined B-cell and plasma cell–depleting strategy. One patient was treated in 2021, whereas the remaining patients received treatment in 2024 and 2025.

This retrospective study was based on routinely collected clinical and biological data. Antinephrin testing was performed retrospectively on stored serum samples from local institutional biobanks at participating centers. All patients provided informed consent for clinical care, including the off-label use of daratumumab, and for use of biological samples for research purposes. In accordance with national regulations for noninterventional retrospective studies, formal institutional review board approval was not required.

### Definitions

Recurrent primary FSGS was defined as the occurrence of persistent nephrotic-range glomerular proteinuria after kidney transplantation, commonly with a urine protein-to-creatinine ratio (UPCR) > 3 g/g, in recipients with a prior history of idiopathic nephrotic syndrome without an identifiable secondary cause in native kidneys. When available, kidney allograft biopsy findings were reviewed to exclude alternative causes of proteinuria; biopsy confirmation was not mandatory in early recurrence.

Response to daratumumab treatment was defined as follows: (i) CR, if the UPCR decreased below 0.5 g/g; (ii) PR, if there was a reduction > 50% in UPCR with residual UPCR < 3 g/g; (iii) no response, defined as the absence of CR or PR, or in case of persistence of apheresis dependence. Patients were considered apheresis-dependent if resumption of apheresis (PEX or IA) for proteinuria control was required within 1 month following the first daratumumab course. Decisions to discontinue apheresis were at the discretion of treating physicians and were not standardized across centers.

Relapse was defined as a new increase in proteinuria > 50% and > 1g/g in ≥ 2 consecutive samples.

### Data Collection

Data were collected retrospectively from hospitalization records, including baseline characteristics, genetic testing, transplantation history, prior therapies, biopsy reports, and daratumumab protocols. For each patient, serial biological parameters were extracted, including UPCR and serum creatinine, to evaluate treatment efficacy. Adverse events associated with daratumumab infusions were also recorded.

### Daratumumab Administration

Because of the absence of established guidelines for daratumumab use in this specific indication, administration protocols (number of doses, timeline and route of administration) varied across centers. All infusions were administered in hospital settings under medical supervision. Subcutaneous daratumumab was administered at the standard dose of 1800 mg/injection in all centers using this route, and i.v. daratumumab was dosed at 16 mg/kg, consistent with hematologic indications. Premedication with dexamethasone and dexchlorpheniramine (or an equivalent antihistamine) was given before each infusion to mitigate infusion-related reactions, and this was consistent across all participating centers.

### Antinephrin Antibodies Testing

Circulating antinephrin IgG were quantified using an enzyme-linked immunosorbent assay. The recombinant extracellular domain of human nephrin (17757-H08H, Sino Biological) was coated onto 96-well plates at a concentration of 200 ng/well in coating buffer (421701, BioLegend) and incubated overnight at 4 °C. The plates were then washed 3 times with 300 μl of phosphate-buffered saline containing 0.05% Tween 20 (0777-1 L, Amresco) and blocked with 300 μl of SuperBlock (37515, Thermo Fisher Scientific) for 1 hour at room temperature. Plasma samples were diluted 1:100 in SuperBlock supplemented with 0.1% Tween 20. One hundred microliters of each diluted sample were added per well and incubated overnight at 4 °C. After 5 washes with phosphate-buffered saline containing 0.05% Tween 20, horseradish peroxidase–conjugated mouse antihuman IgG (ab7499, Abcam) diluted 1:4000 in SuperBlock with 0.1% Tween 20 was added (100 μl/well) and incubated for 1 hour at room temperature with shaking (500 rpm). Following 5 additional washes, 100 μl/well of TMB substrate (421101, BioLegend) was added and allowed to react for 5 minutes at room temperature. The reaction was stopped with 50 μl/well of stop solution (423001, BioLegend), and absorbance was read at 450 nm.

For each sample, net optical density values were calculated by subtracting the mean optical density of antigen-free wells from that of nephrin-coated wells. Antinephrin antibody levels were expressed in relative units (RU/ml), using a standard curve generated from serial 2-fold dilutions of the sample with the highest optical density, arbitrarily set to 1000 RU/ml. The cutoff (98 RU/ml) was defined as the highest value observed among control samples (*n* = 255), a conservative approach used in exploratory biomarker studies to ensure assay specificity. Enzyme-linked immunosorbent assay testing was performed in a blinded fashion with respect to time points and therapy efficacy.

### Outcomes

The primary objective was to assess the efficacy of combined anti-CD38 and anti-CD20 therapy in a multicenter real-world cohort of patients with rFSGS. Secondary objectives included evaluation of safety, detailed clinical and histological characterization of treated patients, and exploratory assessment of longitudinal antinephrin antibody trajectories.

### Statistical Analysis

All statistical analyses and graphical representations were performed using GraphPad Prism (La Jolla, CA; v10.4.2). Continuous variables were described as the median and interquartile range (IQR). Pairwise comparisons of proteinuria and antinephrin antibodies trajectories were performed using the Wilcoxon matched-pairs signed-rank test. Values of *P* < 0.05 were considered significant.

## Results

### Patients’ History

Seventeen kidney transplant recipients with rFSGS were included (Table 1). All patients had a history of idiopathic nephrotic syndrome in their native kidneys at the time of transplantation, except 1 (patient ID06), who had a primary disease of unknown etiology; in this patient, the diagnosis was made retrospectively posttransplant. Three patients, including the latter, had not received immunosuppressive treatment before transplantation. Genetic testing for FSGS-related genes was performed in 7 patients and was negative in all cases (Table 1). Among the 10 patients who were not tested, 6 had undergone a previous kidney transplant, and all experienced disease recurrence, arguing against monogenic forms of FSGS in this subgroup.

**Table 1:** Baseline characteristics of 17 kidney transplant recipients with recurrent FSGS

# ID	Sex	Age at FSGS onset on native kidneys (yrs)	Age at KT (yrs)	Total duration of native disease (yrs)^a^	Genetic testing	Recurrence in previous KT	Type of donor	Residual urine output at KT	Native ureter ligation at KT	Induction treatment	Preventive rituximab at KT	Maintenance immuno-suppression	Time at FSGS recurrence after KT (d)	UPCR at recurrence^b^ (g/g)	Serum creatinine at recurrence (μmol/l)	First line treatment for the recurrence
01	M	62	65	3	Negative	-	DD	Yes	Yes	IL2R-Ab	No	TAC, MPA, CS	1	8.8	181	PEX, CS, RTX
02	M	30	37	3	Negative	-	DD	No		IL2R-Ab	No	CsA, MPA, CS	1	8.2	193	PEX, CS, CsA
03	M	29	50	19	-	-	DD	No		r-ATG	No	TAC, MPA, CS	1	1.8	HD	PEX, CS, RTX, CsA
04	F	54	64	3	Negative	-	LD	No		r-ATG	Yes	TAC, MPA, CS	8	9.1	237	PEX
05	M	40	56	0	-	Yes	LD	No		IL2R-Ab	No	TAC, MPA, CS	238	3.8	160	IA, RTX, CsA
06	F	NA	64	NA	-	-	DD	No		IL2R-Ab	No	TAC, MPA, CS	631	13.0	120	PEX, CS, RTX
07	F	20	44	1	-	Yes	DD	No		r-ATG	No	TAC, MPA, CS	4	27.5	147	PEX, CS, RTX
08	M	20	42	4	-	Yes	LD	Yes	No	IL2R-Ab	Yes	TAC, MPA, CS	2	2.5	191	IA, RTX
09	M	55	58	1	Negative	-	LD	No		IL2R-Ab	No	TAC, MPA, CS	2	2.7	HD	PEX, CS, RTX, CsA
10	F	15	29	8	-	-	LD	No		IL2R-Ab	No	TAC, MPA, CS	4	16.4	282	IA, RTX
11	M	11	29	12	-	Yes	DD	No		r-ATG	Yes	TAC, MPA, CS	16	3.9	80	IA
12	M	16	33	0	-	Yes	DD	No		r-ATG	Yes	TAC, MPA, CS	1456	4.6	90	IA, RTX
13	F	31	50	8	-	Yes	DD	Yes	No	r-ATG	No	CsA, MPA, CS	1	6.4	385	PEX, RTX
14	M	16	23	4	Negative	-	DD	No		r-ATG	No	TAC, MPA, CS	11	3.0	135	IA, CS, RTX
15	F	2	39	33	-	-	DD	No		r-ATG	No	TAC, MPA, CS	3	40.7	183	PEX, IA, RTX
16	M	4	32	11	Negative	-	DD	No		IL2R-Ab	No	TAC, MPA, CS	3	9.0	170	PEX, IA, CS, CsA
17	F	16	24	8	Negative	-	LD	Yes	Yes	IL2R-Ab	Yes	TAC, MPA, CS	55	1.4	130	PEX, CS

CsA, cyclosporine; CS, corticosteroids; Dara, daratumumab; DD, deceased donor; F, female; FSGS, focal segmental glomerulosclerosis; IA, immunoadsorption; IL2R-Ab, basiliximab; KT, kidney transplantation; LD, living donor; M, male; MPA, mycophenolic acid; PEX, plasma exchange; r-ATG, rabbit antithymocyte globulin; RTX, rituximab; TAC, tacrolimus; UPCR, urine protein-to-creatinine ratio.

a From diagnosis to hemodialysis or transplantation if preemptive.

b ± 15 d.

The median age at transplantation was 42 (IQR: 32–50) years, and 10 patients (59%) were male (Table 1). Eleven patients (65%) received a deceased-donor graft. The median time from FSGS diagnosis in the native kidneys to end-stage renal disease (requiring dialysis or transplantation) was 4 (IQR: 2.5–8.8) years. Induction therapy consisted of antithymocyte globulin in 8 patients (47%) and basiliximab in 9 (53%). Maintenance immunosuppression was based on tacrolimus in 15 patients (88%) and cyclosporine in 2 (12%), combined with mycophenolic acid and corticosteroids in all cases.

**Figure 1:**
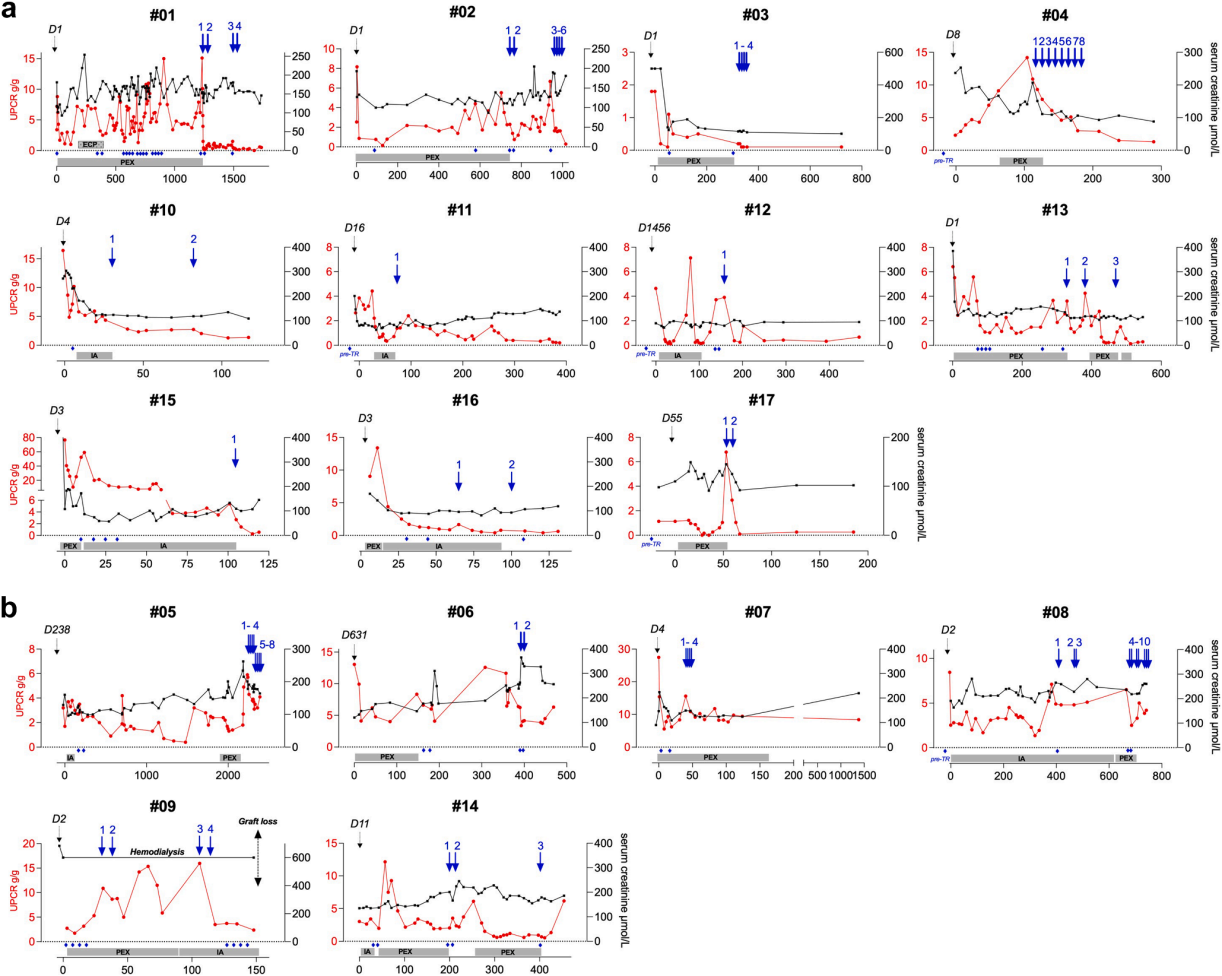
Individual trajectories of proteinuria and serum creatinine after combined anti-CD38 and anti-CD20 therapies in patients with recurrent focal segmental glomerulosclerosis (*N* = 17). Evolution of UPCR, (g/g) and serum creatinine (μmol/l). Each panel represents 1 patient. Responders (complete or partial remission) and nonresponders are shown separately (a and b, respectively). Time 0 corresponds to the diagnosis of recurrent focal segmental glomerulosclerosis, and the time posttransplantation of this diagnosis is indicated in each panel with a black arrow (D, day). Blue arrows indicate daratumumab infusions, grey bars represent apheresis (PEX or IA) or extracorporeal photopheresis and blue diamonds indicate anti-CD20 infusions. IA, immunoadsorption; PEX, plasma exchange; UPCR, urine protein-to-creatinine ratio.

The median time from transplantation to recurrence was 4 (IQR: 2–16) days (Table 1). At recurrence, median proteinuria was 6.4 (IQR: 3–9) g/g and median serum creatinine was 170 (IQR: 133–192) μmol/l; 2 patients required dialysis (Figure 1, Table 1). Graft biopsy was performed in 15 of 17 patients (88%) at recurrence, and in 2 patients after the treatment initiation. All patients received ≥1 line of conventional treatment before daratumumab, including PEX or IA, high-dose corticosteroids, calcineurin inhibitor optimization, and/or rituximab (Figure 1, Table 1). Because of the failure of conventional therapies or the dependency to apheresis, all patients subsequently received a combined rescue strategy incorporating B-cell and plasma cell–targeted therapies (Supplementary Table S1).

### Combined Rescue Therapy

All patients had received B-cell–depleting agents before daratumumab (Table 2). Five patients (29%) received prophylactic rituximab at the time of transplantation, whereas the remaining 12 patients (71%) received anti-CD20 therapy as first- or second-line treatment for FSGS recurrence, with a cumulative exposure ranging from 1 to 15 doses across the cohort. The median time from the first anti-CD20 infusion to daratumumab initiation was 6 (IQR: 3–13) months and the median time from the last anti-CD20 infusion was 21 (IQR: 7–91) days (Table 2, Supplementary Figure S1). Three patients (17%) received an anti-CD20 infusion the same day as the first daratumumab infusion.

**Table 2:** Characteristics of rescue therapy combining daratumumab and anti-CD20 antibodies in rFSGS

# ID	Time from rFSGS to daratumumab (mo)	B-cell-depleting agents before daratumumab (preventive, first- or second-line)	Type of B-cell-depleting agents before daratumumab	Number of B-cell-depleting agent doses before daratumumab	Time between first anti-CD20 and first anti-CD38 (mo)	Time between last anti-CD20 (before anti-CD38) and first anti-CD38 (d)	CD19 count at first daratumumab course (/μl)	Number of daratumumab doses in first course	Total daratumumab doses received
01	40	Yes	OBI, RTX	15	40	0	3	2	4
02	24	Yes	RTX	2	21	0	0	2	6
03	10	Yes	RTX	2	8	15	0	4	4
04	3	Yes	RTX	1	4	147	0	8	8
05	73	Yes	RTX	2	67	2039	28	8	8
06	12	Yes	RTX	3	6	0	0	2	2
07	1	Yes	RTX	2	1	29	0	4	4
08	13	Yes	RTX	1	13	20	41	1	10
09	1	Yes	RTX	4	1	13	0	2	4
10	1	Yes	RTX	1	1	26	0	1	2
11	1	Yes	RTX	1	14	451	0	1	1
12	5	Yes	OBI, RTX	3	52	30	0	1	1
13	10	Yes	RTX	5	7	19	NA	1	3
14	6	Yes	RTX	2	5	1	0	2	3
15	3	Yes	RTX	4	3	74	0	1	1
16	2	Yes	OBI	2	1	21	0	2	2
17	1	Yes	RTX	1	3	108	0	2	2

OBI, obinutuzumab; rFSGS, recurrent focal segmental glomerulosclerosis; RTX, rituximab.

At the time of daratumumab first infusion, 13 patients had complete peripheral B-cell depletion (CD19 = 0/μl), 3 had detectable circulating B cells (ID01, ID05, and ID08), and CD19 data were unavailable in 1 patient (Table 2). Among the 3 patients with incomplete B-cell depletion, 1 (ID01) received an anti-CD20 infusion the same day as daratumumab, whereas the 2 others (ID05 and ID08) did not. Therefore, among the 16 evaluable patients, 14 were effectively under combined B-cell and plasma cell depletion at daratumumab initiation.

### Specificity of Daratumumab Regimen and Concomitant Therapies

Median time from rFSGS to daratumumab initiation was 5 (IQR: 1–10) months (Table 2, Figure 1). Five patients (29%) received daratumumab within the first 2 months following diagnosis (ID07, ID09, ID10, ID11, and ID17). The daratumumab administration protocol, including indication, dosing and infusion schedules, is presented in detail in Supplementary Table S1. Daratumumab was administered subcutaneously in 12 patients (71%) and i.v. in 5 (29%). The median number of doses during the first treatment course was 2 (range:1–8).

At daratumumab initiation, proteinuria was at a median of 3.9 (IQR: 1.9–6.5) g/g (Supplementary Table S2). Apheresis was intensified in 8 patients (47%) during the weeks preceding daratumumab initiation to decrease proteinuria before the initiation (Supplementary Table S2).

Regarding concomitant therapies at treatment initiation, 10 patients (59%) were receiving renin–angiotensin–aldosterone system blockade, and 5 patients (29%) were treated with SGLT2 inhibitors. All patients were receiving mycophenolic acid and a calcineurin inhibitor, including cyclosporine in 6 patients (35%) (Supplementary Table S2). Steroid doses ranged from 5 to 60 mg/day and one patient was steroid-free at daratumumab initiation.

### Histological Features at Daratumumab Initiation

To explore pathological features potentially influencing treatment outcomes, we reviewed allograft biopsy reports performed before daratumumab initiation, or closest to treatment initiation (Supplementary Table S3). Biopsies were performed at variable time points before daratumumab (median [IQR]: 1 [0.5–5.5] month), except in 2 patients (ID09 and ID15), for whom the biopsy was obtained after treatment initiation.

On light microscopy, FSGS lesions were absent in most biopsies (9/17) and, when present, involved only a limited proportion of glomeruli, consistent with early recurrence (Supplementary Table S3). Extensive FSGS lesions (≥ 50% of permeable glomeruli) were observed in only 3 patients (ID05, ID06, and ID09). Chronic lesions were generally mild, except in patients ID05 and ID06 who exhibited more than one-third of sclerotic glomeruli and severe interstitial fibrosis and tubular atrophy (Supplementary Table S3). Immunofluorescence staining was negative or showed only nonspecific IgM and/or C3 trapping. Electron microscopy was not available.

### Outcomes

The trajectories of proteinuria and serum creatinine of the 17 patients after the diagnosis of rFSGS are shown in Figure 1. After the first daratumumab course, CR was achieved in 7 patients (41%) and PR in 4 patients (24%) (Table 3). All responders achieved apheresis independence, as defined by the absence of the need for new apheresis sessions within 1 month following the first daratumumab infusion. Discontinuation occurred in most cases directly after the first daratumumab infusion (Table 3). The median time to response was 24 (IQR: 14–55) days. Two of the 4 patients initially achieving PR subsequently reached CR after a second or third course of daratumumab. Among 9 patients treated with daratumumab within 6 months of recurrence, 7 (78%) achieved CR or PR, compared with 4 of 8 patients (50%) treated later. At the end of follow-up, 6 patients (35%) did not respond.

**Table 3:** Outcomes of combination therapy in rFSGS

# ID	Time from rFSGS to anti-CD38 (mo)	Response to first-line anti-CD38 treatment	Time to response (d)	Apheresis discontinuation after first-line	Time from anti-CD38 to PEX/IA cessation (d)	Relapse after treatment^a^	If relapse, time to relapse (mo)	Follow-up duration after anti-CD38 (mo)	Serum creatinine at last follow-up (μmol/l)	UPCR at last follow-up (g/g)
01	40	CR	6	Yes	0	Yes	8	16	146	0.5
02	24	PR	8	Yes	0	Yes	6	8	181	0.3
03	10	CR	13	Yes	0	No		13	101	0.1
04	3	PR	177	Yes	0	No		5	88	1.3
05	73	No		Yes	0			5	164	4.1
06	12	No		No				2	256	6.3
07	1	No		No				43	220	8.4
08	13	No		No				10	260	4.2
09	1	No		No				4	Graft loss	
10	1	PR	73	Yes	0	No		2	109	1.4
11	1	CR	328	Yes	0	No		10	137	0.2
12	5	CR	36	Yes	0	No		9	95	0.6
13	10	PR	27	Yes	0	Yes	2	8	114	0.8
14	6	No		Yes	0			8	186	7.3
15	3	CR	14	Yes	14	No		1	146	0.7
16	2	CR	24	Yes	28	No		2	119	0.6
17	1	CR	14	Yes	0	No		6	102	0.3

CR, complete remission; IA, immunoadsorption; PEX, plasma exchange; PR, partial remission; rFSGS, recurrent focal segmental glomerulosclerosis; UPCR, urine protein-to-creatinine ratio.

a Relapse was defined as a new increase in proteinuria > 50% and > 1 g/g in ≥2 consecutive samples.

During a median follow-up of 8 (IQR: 4–10) months after daratumumab, relapse occurred in 3 patients (patient ID01, ID02, and ID13) who had previously achieved PR or CR, with a median time to relapse of 6 months (Table 2, Figure 1). All were successfully retreated by a new daratumumab course:1 patient received daratumumab alone (ID13) whereas 2 patients received it in combination with anti-CD20 therapy (ID01 and ID02).

**Figure 2:**
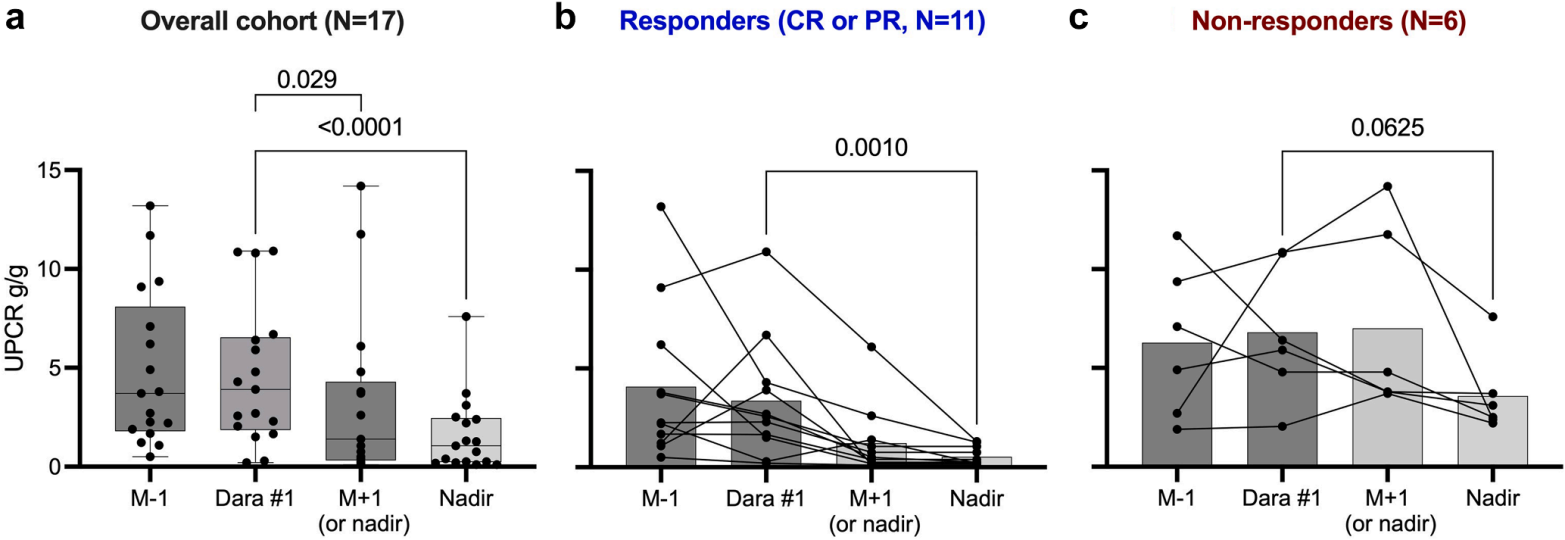
Overall proteinuria response to daratumumab in recurrent focal segmental glomerulosclerosis. (a) Boxplots showing median and interquartile range of UPCR (g/g) in the whole cohort (*N* = 17): 1 month (M−1) before treatment (usually before apheresis intensification), at daratumumab initiation, 1 month (M+1) after initiation, and at proteinuria nadir post–first course. *P*-values from Wilcoxon matched-pairs signed-rank test. Each dot represents one patient. (b) Bar charts showing mean UPCR (g/g) in responders (CR or PR, *n* = 11) at the same time points. Individual patient trajectories are shown as connected lines. *P*-value from Wilcoxon matched-pairs signed-rank test. (c) Bar charts showing mean UPCR (g/g) in nonresponders (*n* = 6) at the same time points. Individual patient trajectories are shown as connected lines. CR, complete remission; PR, partial remission; UPCR, urine protein-to-creatinine ratio.

Among the overall cohort, proteinuria decreased significantly from a median of 3.9 (IQR: 1.9–6.5) g/g at daratumumab initiation to 1.4 (IQR: 0.3–4.3) g/g at 1 month post–first dose (*P* = 0.029) (Figure 2a, Supplementary Table S1). The effect was even more pronounced when considering proteinuria nadir post–first course, with a median of 1.06 (IQR: 0.2–2.5) g/g (*P* < 0.0001).

In the responders’ group, including both partial and complete response, proteinuria decreased significantly from a median of 2.6 (IQR: 1.5–4.3) g/g at daratumumab initiation to 0.26 (IQR: 0.2–1.1) g/g at nadir post–first dose (*P* = 0.001) (Figure 2b). There was no significant decrease in proteinuria in the nonresponders: median of 6.2 (IQR: 4.1–10.8) g/g at daratumumab initiation to 2.8 (IQR: 2.4–4.7) g/g at nadir post–first dose (Figure 2c).

At last follow-up, median serum creatinine was 141 (IQR: 107–182) μmol/l and median proteinuria was 0.7 (IQR: 0.4–2.75) g/g. One patient who had remained on dialysis throughout ultimately lost the graft.

### Treatment Tolerability

Daratumumab was overall well-tolerated. Seven patients experienced nonserious infusion-related reactions and 3 developed cytopenia. One patient experienced a transient myopic shift 1 day after his third injection. This rare side effect has previously been documented with daratumumab; and as reported in the literature, his symptoms resolved spontaneously, allowing therapy to be continued. Seven patients (41%) experienced hypogammaglobulinemia requiring prophylactic i.v. Ig supplementation. One patient presented a nonsevere urinary tract infection without hypogammaglobulinemia.

### Antinephrin Antibody Trajectories

**Figure 3:**
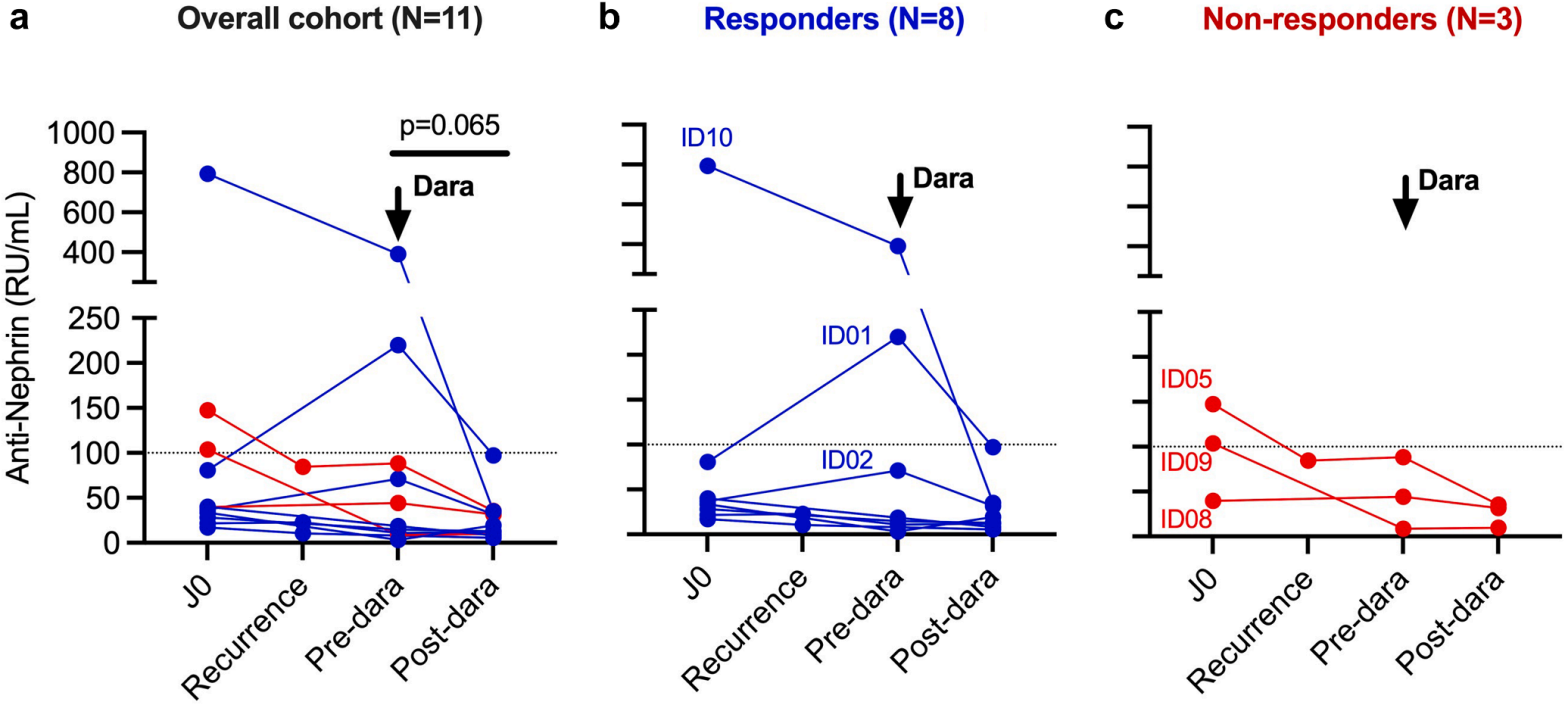
Longitudinal evolution of antinephrin antibody levels following daratumumab treatment. (a) Individual trajectories of antinephrin antibody levels are shown for the 11 of 17 patients in whom measurements were available. Time points include pretransplant baseline, recurrence, pre-daratumumab, and post-daratumumab assessments, with detailed sample characteristics provided in Supplementary Table S3. Patients were classified as (b) responders (blue) or (c) nonresponders (red) according to the predefined criteria (see Methods). Daratumumab administration is indicated by an arrow. Results are expressed in relative units and the positivity threshold, defined as the highest value observed in healthy controls, is shown as a dotted horizontal line (98 RU/ml).

Antinephrin IgG levels were retrospectively assessed in 11 of 17 patients with available serum samples at transplantation (day 0), recurrence, and before and after daratumumab treatment (Figure 3; Supplementary Table S4). Three patients had antibody levels above the predefined positivity threshold (98 RU/ml) at transplantation. Two patients were positive immediately before daratumumab initiation and demonstrated marked posttreatment reductions in antibody titers that paralleled clinical improvement. In contrast, 1 patient had elevated antinephrin antibodies at the time of transplantation, with titers that declined spontaneously over time and did not show a clear response to daratumumab. Notably, in patient ID01, antibody titers incraesed again at relapse and subsequently declined following daratumumab re-treatment (Supplementary Table S4).

## Discussion

In this multicenter retrospective cohort, combined anti-CD38 and anti-CD20 therapy was associated with meaningful remission rates in patients with resistant rFSGS. More than half of patients reached CR or PR, and all responders were able to discontinue apheresis. In addition, longitudinal antinephrin antibody trajectories provided exploratory biological insight into treatment response.

rFSGS remains a significant burden after kidney transplantation, often refractory to first-line treatments, with persistent proteinuria driving allograft dysfunction and prolonged apheresis dependence, substantially reducing quality of life.^3^^,^^7^ The combined anti-CD20/anti-CD38 approach was initially reported in multidrug-resistant and multidrug-dependent nephrotic syndromes on native kidneys,^15^^,^^16^ providing the rationale for its application in the posttransplant setting. Indeed, rFSGS represents a unique clinical scenario in which the immune-mediated nature of the disease is established by the very fact of recurrence in a nonaffected allograft, making it an ideal setting to evaluate the efficacy of combined B-cell and plasma cell depletion and to gain insight into the pathogenesis of this disease.

In our cohort, approximately 60% of patients achieved remission, echoing the efficacy signal reported in prior case series and in a recent multicenter study using combined anti-CD20 and anti-CD38 therapy. Angeletti *et al.*^17^ reported successful treatment in 5 patients and Randone *et al.*^19^ described sustained remission in 2 additional cases. Finally, a recent retrospective study reported high response rates with combined anti-CD20 and anti-CD38 therapy, with 15 of 16 patients achieving CR or PR, although a subset of responder patients remained dependent on apheresis at last follow-up.^20^ In our cohort, a subset of patients failed to respond. Among nonresponders, 2 patients showed more advanced chronic lesions on biopsy before treatment (ID05 and ID06) than the rest of the cohort, which may have limited reversibility. In addition, patient ID09 presented with particularly severe early disease, remained dialysis-dependent, and ultimately lost the graft. Relapses occurred in 27% of responders, indicating that a single treatment course may not ensure durable disease control; as reported previously,^17^ relapses responded to retreatment.

Factors influencing response or relapse are difficult to determine in a small retrospective cohort, but several observations emerge. First, all patients had received previous anti-CD20 therapy, and 14 of 17 were effectively under combined B-cell and plasma cell depletion at daratumumab initiation, as reflected by complete CD19 depletion in 13 cases (reflecting the prolonged pharmacodynamic effect of anti-CD20 therapy^21^) and same-day anti-CD20 administration in 1 additional case. Although the time interval between the last anti-CD20 infusion and daratumumab initiation varied considerably (from days to years), no clear association with treatment response was observed, with overlapping distributions between responders and nonresponders (Supplementary Figure S1). Notably, the 2 patients with incomplete B-cell depletion who did not receive same-day anti-CD20 (ID05 and ID08) were both nonresponders, suggesting that effective combined B-cell and plasma cell depletion may be important for treatment response, although this observation is based on only 2 cases. Thus, collectively, our data do not allow attribution of efficacy to anti-CD38 alone but rather support a combined plasma cell– and B-cell–depleting strategy. Association of anti-CD20 antibodies with daratumumab may help sustain remission by depletion of memory B cells. Of note, as previously reported, some relapses responded to anti-CD38 retreatment alone, suggesting a sustained role for anti-CD38 in selected patients.^17^ Conversely, the time to response varied considerably across patients, and in some cases the progressive decline in proteinuria may reflect the combined effect of daratumumab, ongoing immunosuppression, and prior anti-CD20 therapy.

Second, antinephrin antibodies were assessed in 11 patients and were positive in only a minority of cases, which may partly reflect concomitant immunosuppressive treatment at the time of sampling. In the 2 patients who were positive before daratumumab treatment, antibody levels paralleled clinical response, and in the patient who relapsed and subsequently responded to retreatment, antibody levels again tracked with the clinical course. However, these individual trajectories, though suggestive, do not establish a consistent relationship between antibody status and treatment response across the cohort. Importantly, the absence of antinephrin antibodies does not exclude the involvement of other autoantibodies directed against slit diaphragm proteins^22^ or non–antibody-mediated pathogenic mechanisms that could nonetheless respond to anti-CD38 depletion. These findings therefore remain exploratory and are not yet sufficient to establish a pathogenic or classificatory role for antinephrin antibodies in this setting.

Third, unlike earlier reports,^18^ PEX was not consistently intensified before daratumumab treatment, which may have reduced efficacy in some patients given the risk of drug loss through heavy proteinuria. Finally, treatment regimens varied widely across centers, reflecting real-world practice in the absence of a codified therapy. Daratumumab was administered at variable time points and doses, and some patients required repeated treatment whereas others responded after 1 or 2 doses (ID11 and ID12).

Collectively, the optimal dosing schedule of anti-CD38, as well as the role of combined B-cell depletion and biomarker-guided retreatment strategies, remain to be defined.

The optimal timing of daratumumab initiation remains to be determined. In our cohort, the combined anti-CD38 and anti-CD20 strategy was primarily used as rescue therapy and demonstrated the ability to induce remission in highly refractory patients. Although overall remission rates were comparable with those reported with conventional first-line strategies such as those described by Uffing *et al.*^3^ (∼ 60%) and Lanaret *et al.*^5^ (∼75%), the capacity of this approach to achieve remission in patients who had failed standard treatments, with subsequent discontinuation of apheresis, supports its role as an effective rescue option. Importantly, the burden of prolonged apheresis—a time-consuming and quality-of-life–limiting therapy—is greatly alleviated by a short course of antibody injections. The rapid and marked responses observed in some patients raise the possibility of benefit if introduced earlier in high-risk cases. In a descriptive analysis without formal statistical comparison, we observed a higher remission rate among patients treated within 6 months of recurrence (78%) compared with those treated later (50%), consistent with the hypothesis that early intervention may help prevent irreversible podocyte injury. However, 2 patients treated early (ID07 and ID09) did not respond, and the small sample size and treatment heterogeneity preclude firm conclusions. Our retrospective data do not allow conclusions regarding its use as first-line therapy.

Therefore, based on our observations and prior reports, we propose a hypothesis-generating treatment framework to inform future prospective trials (Supplementary Figure S2).

Regarding safety, daratumumab was generally well-tolerated. Mild infusion-related reactions were observed, and 1 patient presented a transient myopic shift, previously described after daratumumab.^23^ However, more than one-third developed hypogammaglobulinemia requiring i.v. Ig, a concern amplified by the heavy baseline immunosuppression and prior anti-CD20 exposure in this population. Considering the increased infection risk associated with daratumumab in hematologic settings,^24^ systematic monitoring of gammaglobulin levels and early prophylactic i.v. Ig are recommended, and longer follow-up is needed to fully characterize the safety profile.

This study has limitations. The small sample size and retrospective design introduced considerable heterogeneity in treatment regimens, including differences in the number of daratumumab doses, timing of administration, route of administration, and concomitant therapies. Decisions to discontinue apheresis were not standardized and may have been influenced by center-specific practices. Although all these reflect real-world practice in the absence of established guidelines, it limits generalizability and precludes firm conclusions regarding predictors of response. Formal exploratory subgroup analyses were not performed because of the high risk of underpowered and misleading results. The relatively short follow-up duration prevented the evaluation of long-term efficacy and safety. Longitudinal monitoring of CD38^+^ cell subset was not available and should be incorporated into future prospective trials because it might influence response kinetics. Genetic testing for FSGS-associated variants was performed in only a subset of patients. Although recurrence after transplantation and recurrence in previous grafts may argue against monogenic forms of disease, the absence of systematic genetic testing remains a limitation. Finally, electron microscopy was not routinely performed in our centers, limiting ultrastructural assessment of podocyte injury.

In conclusion, our findings support a combined plasma cell- and B cell-depleting strategy as a promising rescue approach for rFSGS after kidney transplantation. In patients refractory to standard of care, this combined strategy induced rapid remission and enabled discontinuation of apheresis with an acceptable safety profile. Prospective trials are needed to define optimal dosing strategies, long-term safety, predictors of response, and the place of this combined approach within the therapeutic algorithm for rFSGS.

## Disclosure

All the authors declared no competing interests.

## Supplementary Material

Supplementary File (PDF) Figure S1. Time interval between the last anti-CD20 infusion and daratumumab initiation, stratified by treatment response. Figure S2. Proposal treatment framework for recurrent FSGS after kidney transplantation. Table S1. Daratumumab (anti-CD38) administration protocols by center in recurrent FSGS. Table S2. UPCR and concomitant medications at daratumumab initiation. Table S3. Histological characteristics of the closest graft biopsy to the combination therapy. Table S4. Serum samples characteristics of antinephrin testing antibody.
