# Supplementary material for: Anti-CD38/Anti-CD20 Rescue Therapy for Posttransplant Recurrent FSGS
Source: Kidney Int Rep. 2026 Jun 16;11(9):106668. doi: 10.1016/j.ekir.2026.106668 (PMC13452287; doi:10.1016/j.ekir.2026.106668)
Supplement: Supplementary file 1 [file mmc1.pdf]

Supplementary Material

Supplementary Figure Legends

Figure S1. Time interval between the last anti-CD20 infusion and daratumumab initiation, stratified by treatment response.

Bar charts showing the mean time (days) from the last anti-CD20 infusion to the first anti-CD38 infusion in complete responders (CR, n=7), partial responders (PR, n=4), and non-responders (NR, n=6). Each dot represents one patient. Star symbols indicate the two patients with incomplete B-cell depletion who did not receive same-day anti-CD20 (ID05 and ID08).

Figure S2. Proposal treatment framework for recurrent FSGS after kidney transplantation.

This diagram illustrates a proposed management framework for post-transplant recurrent FSGS, derived from the present cohort and prior reports. After diagnosis based on glomerular proteinuria and kidney biopsy excluding alternative causes, patients receive standard first-line therapy including corticosteroids, intensive apheresis, optimization of calcineurin inhibitor (CNI) exposure, and anti-CD20 therapy when not previously administered. In the absence of adequate response, rescue therapy with combined plasma cell (anti-CD38) and B-cell (anti-CD20) depletion may be considered. The framework also highlights pragmatic considerations for retreatment, safety monitoring (including hypogammaglobulinemia), and exploratory biomarker assessment. This figure is intended to inform future prospective study design and does not represent a validated treatment recommendation.

D, day; FSGS, focal segmental glomerulosclerosis; IA, immunoadsorption; IVIg, intravenous immunoglobulin; PEX, plasma exchange; UPCR, urine protein-to-creatinine ratio;

Supplementary Table Legends

Table S1. Daratumumab (anti-CD38) Administration Protocols by Center in Recurrent FSGS

CR : Complete Remission IV: intravenous; PR : Partial Remission; SC: subcutaneous
* **Rescue** : after 2 or more treatment lines

Table S2. UPCR and concomitant medications at daratumumab initiation

ACEi: angiotensin-converting enzyme inhibitor; ARB: angiotensin II receptor blocker ; CNI, calcineurin inhibitor; CsA: cyclosporine; MPA: mycophenolic acid; RAAS: renin–angiotensin–aldosterone system; rFSGS, recurrent focal segmental glomerulosclerosis; SGLT2: sodium–glucose cotransporter-2; TAC: tacrolimus; UPCR: urine protein-to-creatinine ratio

Table S3. Histological characteristics of the closest graft biopsy to the combination therapy

CR: complete remission; PR: partial remission; NA, no available; rFSGS, recurrent focal segmental glomerulosclerosis
*Biopsy performed after treatment (no biopsy before treatment initiation)

Table S4. Serum samples characteristics of anti-nephrin testing antibody

CR: complete remission; dara: daratumumab PR: partial remission; TR: transplantation

Figure S1

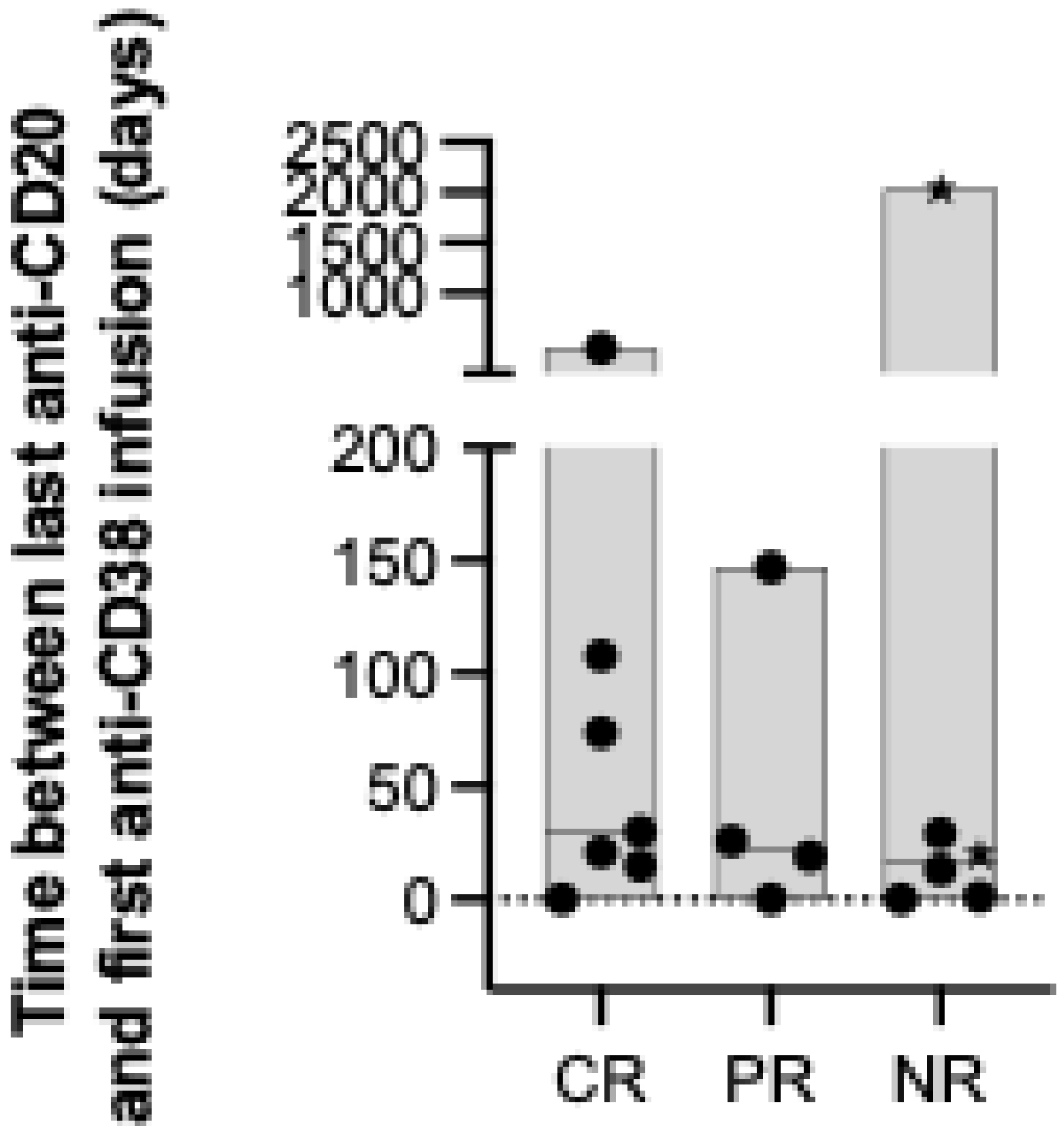
Time between last anti-CD20
and first anti-CD38 infusion (days)
2500
2000
1500
1000
200
150
100
50
0
CR
PR
NR

Figure S2

Post-transplant FSGS recurrence
Glomerular proteinuria + Kidney biopsy excluding other causes

↓

"Standard" Treatment for 2-4 weeks
- 3 steroid pulses followed by tapering
- Intensive apheresis (PEX or IA, >= 3 sessions per week)
- Optimization of calcineurin inhibitor exposure

+/- Anti-CD20 if not previously administered prophylactically

If no response (UPCR decreases <50% and/or >1g/g):

↓

Rescue therapy = combined anti-CD20 and anti-CD38 depletion

2 sub-cutaneous infusions of 1800mg a week apart (D0 and D7)
Combined with an anti-CD20 infusion at Day 0 if not previously administered or if CD19+ B cells >1/µL

If feasible: intensify apheresis before the first infusion to reduce proteinuria, then discontinue after the first infusion
If failure at Day 14 after the first daratumumab (UPCR decreases <50% and/or > 1g/g): intensify treatment with 2 additional infusions at D14 and D21
If relapse (sustained UPCR > 0.5 g/g) : 2 new infusions of anti-CD38 a week apart +/- anti-CD20 if CD19$^+$ B cells >1/µL
Monitor serum protein electrophoresis: if hypogammaglobulinemia < 5g/L → IVIg supplementation
Exploratory monitoring of anti-nephrin antibodies and CD38+ cells : Baseline and follow-up

Table S1. Daratumumab (anti-CD38) Administration Protocols by Center in Recurrent FSGS

# ID	Center Name	Indication for Initiation	Number of anti-CD38 infusions in first course	Interval between anti-CD38 infusions in first course (days)	Route of anti-CD38 infusion	Dose per infusions	Time between last anti-CD20 and first anti-CD38 (days)
01	Necker Hospital. Paris	Rescue* : multiresistant with apheresis dependency	2	7	SC	1800 mg	0
02	Bicêtre Hospital, Paris	Rescue : multiresistant with apheresis dependency	2	7	SC	1800 mg	0
03	Tours University Hospital	2nd line : apheresis dependency	4	7	SC	1800 mg	15
04	Tours University Hospital	2nd line : resistance to first line	8	7	SC	1800 mg	147
05	Tours University Hospital	Rescue : multi-resistant with apheresis dependency	8	7	SC	1800 mg	2039
06	Georges Pompidou Hospital, Paris	Rescue : multiresistance	2	10	SC	1800 mg	0
07	Henri-Mondor Hospital, Créteil	2nd line : resistance to first line	4	7	IV	16 mg/kg (700 mg)	29
08	Rangueil Hospital, Toulouse	2nd line : resistance with apheresis dependency	1	--	SC	1800 mg	20
09	Reims University Hospital	2nd line : resistance with apheresis dependency	2	7	SC	1800 mg	13
10	Rouen University Hospital	2nd line : PR with apheresis dependency	1	--	IV	16 mg/kg (990 mg)	26
11	Rouen University Hospital	Consolidation: CR with apheresis dependency	1	--	IV	16 mg/kg (720 mg)	451
12	Rouen University Hospital	2nd line : Recurrence after spacing apheresis	1	--	IV	16 mg/kg (1200 mg)	30
13	Caen University Hospital	Rescue : multiresistant with apheresis dependency	1	--	IV	16 mg/kg (1480 mg)	19
14	Lapeyronie Hospital, Montpellier	Rescue : multiresistant with apheresis dependency	2	7	SC	1800 mg	1
15	Félix-Guyon Hospital, La Réunion	Rescue : multiresistant with apheresis dependency	1	--	SC	1800 mg	74
16	Hospices Civils de Lyon, Lyon	2nd line : Recurrence after spacing apheresis	2	31	SC	1800 mg	21
17	Necker Hospital. Paris	2nd line : Recurrence after spacing apheresis	2	7	SC	1800 mg	108

CR : Complete Remission IV: intravenous; PR : Partial Remission; SC: subcutaneous
* ***Rescue*** *: after 2 or more treatment lines*

Table S2. UPCR and concomitant medications at daratumumab initiation

# ID	Time from rFSGS to daratumumab (months)	Intensive plasmapheresis before daratumumab initiation	UPCR within a month before daratumumab initiation (g/g)	UPCR at daratumumab initiation (g/g)	RAAS Blockade	SGLT2 inhibitor	CNI	Anti-proliferative	Steroid Dose (mg/day)
01	40	Yes	6.2	1.5	ACEi, ARB	Yes	CsA	MPA	10
02	24	Yes	2.3	2.3	ACEi	Yes	CsA	MPA	10
03	10	Yes	0.5	0.2	None	No	CsA	MPA	10
04	3	No	9.1	10.9	ARB	No	TAC	MPA	10
05	73	No	4.9	5.9	ACEi	Yes	CsA	MPA	10
06	12	No	11.7	6.4	ACEi	No	CsA	MPA	10
07	1	Yes	9.4	10.9	ARB	No	TAC	MPA	40
08	13	No	7.1	4.8	None	No	TAC	MPA	5
09	1	Yes	2.7	10.8	None	No	CsA	MPA	60
10	1	Yes	13.2	4.3	None	No	TAC	MPA	10
11	1	Yes	2.2	0.3	None	No	TAC	MPA	10
12	5	No	1.1	3.9	None	No	TAC	MPA	10
13	10	No	3.7	2.6	ARB	Yes	TAC	MPA	None
14	6	No	1.9	2.0	ACEi, ARB	Yes	TAC	MPA	10
15	3	No	3.8	2.7	None	No	TAC	MPA	5
16	2	Yes	1.7	1.7	ARB	No	TAC	MPA	30
17	1	No	1.2	6.7	ARB	No	TAC	MPA	60

ACEi: angiotensin-converting enzyme inhibitor; ARB: angiotensin II receptor blocker ; CNI, calcineurin inhibitor; CsA: cyclosporine; MPA: mycophenolic acid; RAAS: renin–angiotensin–aldosterone system; rFSGS, recurrent focal segmental glomerulosclerosis; SGLT2: sodium–glucose cotransporter-2; TAC: tacrolimus; UPCR: urine protein-to-creatinine ratio

Table S3. Histological characteristics of the closest graft biopsy to the combination therapy

# ID	Time from rFSGS to daratumumab (months)	Response to first line daratumumab treatment	Time between biopsy and daratumumab (months)	Total glomeruli	Permeable glomeruli (% of total glomeruli)	FSGS lesions (% of permeable glomeruli)	Chronic lesions (ct and/or ci score)	Immunofluorescence staining
01	40	CR	0	12	83%	0%	2	negative
02	24	PR	21	9	100%	15%	0	negative
03	10	CR	4	29	93%	0%	2	IgM traces & C3 traces
04	3	PR	3	47	97%	4%	1	IgM traces & C3 traces
05	73	No	1	3	63%	50%	3	IgM traces & C3 traces
06	12	No	0	19	68%	50%	3	IgM traces & C3 traces
07	1	No	1	21	90%	0%	1	negative
08	13	No	11	31	97%	13%	0	negative
09	1	No	1 month later*	19	89%	59%	0	negative
10	1	PR	0	4	75%	0%	1	IgM traces & C3 +
11	1	CR	1	13	100%	0%	1	IgM traces & C3 traces
12	5	CR	5	12	92%	36%	1	IgM traces
13	10	PR	10	0 (inadequate)	NA	NA	NA	NA
14	6	No	6	7	100%	0%	0	negative
15	3	CR	3 month later*	20	90%	0%	1	NA
16	2	CR	0	8	100%	0%	0	IgM traces & C3 traces
17	1	CR	1	12	75%	0%	0	IgM traces

CR: complete remission; PR: partial remission; NA, no available; rFSGS, recurrent focal segmental glomerulosclerosis
*Biopsy performed after treatment (no biopsy before treatment initiation)

Table S4. Serum samples characteristics of anti-nephrin testing antibody

# ID	Serum ID	Center Name	Time-point(s)	Time post-transplantation (days)	Time post-daratumumab (days)	Anti-Nephrin ELISA value (RU/mL)	Anti-Nephrin positive	Outcome after first daratumumab course
ID01	25	Necker (Paris)	D0	0		80.6	0	CR
	33		Pre-dara	1206		219.8	**1**	
	34		Post-dara	1261	20	97.1	0	
	35		Pre-dara (relapse)	1492		128.7	**1**	
	36		Post-dara (after re-treatment)	1512	20	80.8	0	
ID02	55	Bicetre (Paris)	D0	0		37.1	0	PR
	56		Pre-dara	962		71.2	0	
	57		Post-dara	976	7	31.5	0	
ID03	52	Tours	D0	15 day pre-TR		16.9	0	CR
	53		Recurrence / Pre-dara	1		10.4	0	
	54		Post-dara	356	34	5.7	0	
ID04	48	Tours	D0	0		21.8	0	PR
	49		Recurrence	8		22.7	0	
	50		Pre-dara	112		10.9	0	
	51		Post-dara	162	42	12.1	0	
ID05	44	Tours	D0	0		147.5	**1**	No response
	45		Recurrence	188		84.4	0	
	46		Pre-dara	2430		88.5	0	
	47		Post-dara	2535	62	35.3	0	
ID08	72	Toulouse	M-1 (Rituximab)	30 day pre-TR		64.3	0	No response
	73		D0	0		39.8	0	
	74		Pre-dara	82		44.3	0	
	75		Post-dara	820	77	31.7	0	
ID09	39	Reims	D0	0		103.7	**1**	No response
	40		Pre-dara	16		8.6	0	
	41		Post-dara #1	93	53	9.7	0	
	42		Post-dara #2	151	111	14.9	0	
ID10	58	Rouen	D0	0		793.3	**1**	PR
	59		Pre-dara	16		391.5	**1**	
	60		Post-dara #1	44	9	35.9	0	
	61		Post-dara #2	91	56	35.1	0	
ID11	62	Rouen	D0	0		39.9	0	CR
	63		Pre-dara	45		18.8	0	
	64		Post-dara #1	95	19	8.2	0	
	65		Post-dara #2	234	158	15.1	0	
	66		Post-dara #3	366	290	42.2	0	
ID12	67	Rouen	D0	0		28.1	0	CR
	68		Pre-dara	1469		14.6	0	
	69		Post-dara #1	1750	120	13.0	0	
	70		Post-dara #2	1851	221	8.1	0	
	71		Post-dara #3	2015	385	11.1	0	
ID17	32	Necker (Paris)	D0	0		33.4	0	CR
	37		Pre-dara	108		3.1	0	
	38		Post-dara	240	132	19.3	0	

CR: complete remission; dara: daratumumab PR: partial remission; TR: transplantation
